# X-ray and Neutron Diffraction Studies of SrTe_2_FeO_6_Cl, an Oxide Chloride with Rare Anion Ordering

**DOI:** 10.1021/acs.inorgchem.3c01951

**Published:** 2023-08-02

**Authors:** Johnny A. Sannes, Bruno Gonano, Øystein S. Fjellvåg, Susmit Kumar, Ola Nilsen, Martin Valldor

**Affiliations:** † Centre for Materials Science and Nanotechnology (SMN), Department of Chemistry, University of Oslo, Sem Sælands vei 26, Oslo N-0371, Norway; ‡ Department for Hydrogen Technology, Institute for Energy Technology, Kjeller NO-2027, Norway; § Laboratory for Neutron Scattering and Imaging, Paul Scherrer Institute, Forschungsstrasse 111, Villigen PSI 5232, Switzerland

## Abstract

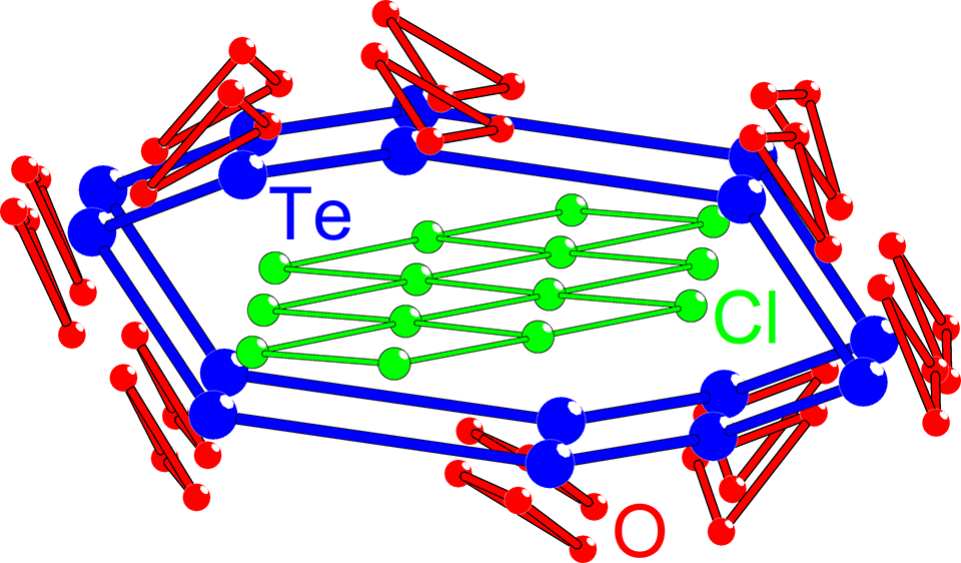

The oxychloride SrTe_2_FeO_6_Cl is obtained by high-temperature solid-state synthesis under inert conditions in closed reaction vessels. The compound crystallizes in a novel monoclinic crystal structure that is described in the space group *P*12_1_/*n*1 (No. 14). The unit cell parameters, *a* = 10.2604(1) Å, *b* = 5.34556(5) Å, *c* = 26.6851(3) Å, and β = 93.6853(4)°, and atomic parameters were determined from synchrotron diffraction data, starting from a model that was obtained from single-crystal X-ray diffraction data. The anion lattice exhibits a rare ordering of oxide and chloride ions: one-dimensional zig-zag ladders of chlorine (squarelike motif) are surrounded by an oxygen matrix. Two different iron sites coordinated solely to oxygen are present in the structure, one octahedral and one square pyramidal, both distorted. Similarly, two different strontium coordinations are present; the first homoleptic coordinated to eight oxygen atoms and the second heteroleptic coordinated to four oxygen and four chlorine atoms in a *fac*-like manner. The lone pair of Te(IV) is directed toward the larger chlorine atoms. Magnetic susceptibility measurements confirm that Fe is +3 (*d*^5^) in the high-spin electronic configuration, exhibiting an almost ideal spin-only moment, μ_eff_ = 5.65 μ_B_ Fe^–1^. The slightly negative Weiss constant (θ_CW_ = −39 K) suggests dominating antiparallel spin-to-spin coupling in the paramagnetic temperature range, agreeing with an observed long-range antiferromagnetic spin ordering below Néel temperature, *T*_N_ ∼ 13 K, and a broad second order-like anomaly in the specific heat measurement data. Low-temperature neutron diffraction data reveal that the antiferromagnetic ordered phase is C-type, with a *k*-vector (1/2, 1/2, 0) and ordered moment of 4.14(7) μ_B_. The spin structure can be described as antiferromagnetic ordered layers stacked along the *a*-axis, forming layers of squares that alternate along the *c*-axis.

## Abstract 2

An oxychloride, SrTe_2_FeO_6_Cl, with a novel crystal structure is discovered and the structure was solved by synchrotron diffraction. A rare anion ordering is observed; zig-zag ladders of chlorine are surrounded by lone pair active tellurium and oxygen. At low temperatures, an antiferromagnetic spin structure forms and was investigated by neutron diffraction, thereby solving the spin structure.

## Introduction

Mixed anion compounds have gained attention in recent years because they represent a relatively new way of designing structural chemistry, including crystal fields, band gaps, and local symmetries.^1,2^ Typical examples of combining oxide and chloride chemistries result in novelties such as Cu_2_Te_2_O_5_X_2_ (X = Cl and Br),^3^ which contain Cu(II), a *d*^9^ ion with low-dimensional quantum spin and Co_2_TeO_3_Cl_2_,^4^ a layered structure with antiferromagnetic (AFM) ordering. The introduction of multiple anions also leaves the possibility for anion ordering in multiple dimensions. This phenomenon is, for instance, observed in Ba_4_OCl_6_,^5^ CsCrO_3_Cl,^6^ and SmSb_2_O_4_Cl,^7^ which has 0D, 1D, and 2D anion ordering, respectively. The introduction of multiple anions can also lead to the formation of Janus materials, which may be of interest for their thermoelectric properties^8^ or for photocatalytic water splitting^9^ applications.

The use of p-block elements with stereochemically active lone pairs like Sn(II), Pb(II), Sb(III), Bi(III), Se(IV), and Te(IV) together with halide ions have gained much popularity as it often leads to novel low-dimensional structures.^3,4^ This concept has been expanded upon by including large alkaline-earth metals to reduce the structural dimensionality.^10^ Combining an ion with an active lone pair in a lattice containing more than one anion is a possible route to finding novel compounds capable of addressing society’s most urgent technological needs. Moreover, an open question is how the lone pair will act in a mixed coordination environment.

If magnetic ions are integrated into the crystal lattice with two anions and a lone pair ion, local and global polarities could affect the magnetic properties, which is interesting for both fundamental studies and application purposes. An example of a possible application is using single layers of bulk antiferromagnets for spintronics,^11^ and fundamental research still focuses on the correlations between superconductivity and antiferromagnetism.^12^ Herein, SrTe_2_FeO_6_Cl is presented, an oxychloride with an unprecedented crystal structure, where Fe exhibits antiferromagnetic long-range ordering at low temperatures.

## Materials and Methods

Small single crystals of SrTe_2_FeO_6_Cl were observed when reacting SrCl_2_ (Alfa Aesar 99.995%), Fe_2_O_3_ (VWR Chemicals 99%), Fe (Alfa Aesar 99%), and TeO_2_ (Acros Organics 99+%) in stoichiometric amounts corresponding to SrTe_3_Fe_2_O_8_Cl_2_, which is related to already known compounds.^10^ Inside an Ar-filled glovebox (GS Glovebox Systemtechnik GmbH, O_2_ and H_2_O < 2 ppm), stoichiometric amounts of constituents were homogenized by grinding in an agate mortar. The visibly homogeneous powder was filled in an alumina crucible that was loaded into a silica ampule, which was partly evacuated using a septum and syringe inside a glovebox before being subsequently sealed using an oxygen/hydrogen torch. The closed ampule with the sample was heated at 5 °C/min in a muffle furnace up to 600 °C and was kept there for 10 h before being cooled at an ambient rate down to room temperature. After the heat treatment, small metallic particles, most likely metallic Te, were observed at the top of the ampule.

The SrTe_2_FeO_6_Cl sample, used for further measurements, was synthesized using the conventional high-temperature solid-state synthesis technique under inert conditions. One constituent, SrO, was obtained by thermally decomposing SrCO_3_ (Fluka ≥98%) under dynamic vacuum (<10^–2^ mbar) overnight at 1030 °C. It was transported to an Ar-filled glovebox without exposure to air. The other constituents were the same as described previously: SrCl_2_, Fe_2_O_3_, and TeO_2_. The synthesis was performed as described above; however, the sample was kept at 650 °C for 10 h. This synthesis also led to small crystals of SrTe_2_FeO_6_Cl. To synthesize enough material for neutron measurements, a total of 4 g of the constituents was mixed in stoichiometric amounts and roughly divided into four crucibles and prepared following the procedure described above.

### Powder and Single-Crystal X-ray Diffraction

Powder X-ray diffraction (pXRD) results were obtained using a Bruker D8 DISCOVER with Bragg–Brentano geometry equipped with a Ge(111) Johanssen monochromator, Cu*K*α_1_ (λ = 1.5406 Å) and a LYNXEYE detector. A flat plate, fitted with a glass plate and coated with silicone grease, was used as the sample holder. Single-crystal XRD (SC-XRD) data were obtained with a Bruker D8 VENTURE single-crystal diffractometer. The radiation source was a Mo *K*_α_ InCoatec microfocus X-ray source using a PHOTON 100 detector. The structure determination and refinement were done using JANA2020.^13^

### Scanning Electron Microscopy and Energy-Dispersive X-ray Spectroscopy

Scanning electron microscopy (SEM) images and the elemental composition were determined using a Hitachi SU8230 field-emission scanning electron microscope (FESEM) and an XFlash 6|10 energy-dispersive X-ray spectroscopy (EDX) detector, respectively. Images were captured using an electron acceleration voltage of 5 kV and an electron current of 5 μA. In comparison, the elemental composition was measured using a voltage of 20 kV and an electron current of 30 μA. The average elemental composition was estimated from measurements on 10 different crystallites.

### Physical Property Measurement System

Magnetic and specific heat measurements were performed on a Quantum Design physical property measurement system (PPMS). The magnetic susceptibility was measured using polycrystalline material packed in a polypropylene sample holder held in a brass sample holder, and the measurements were conducted from 2 to 300 K at 0.1 T. The specific heat capacity was measured using a cold-pressed platelet between 2 and 300 K with the standard, nonadiabatic thermal relaxation method and subtracting the addenda from the sample signal.

### Synchrotron Diffraction

Synchrotron pXRD was performed at BM01, SNBL (Swiss-Norwegian Beamlines) at ESRF (European Synchrotron Radiation Facility), using λ = 0.69127 Å. The investigated SrTe_2_FeO_6_Cl sample was carefully ground and introduced to a 0.3 mm silica capillary. Data were collected at RT using a 2D PILATUS 2 M detector at a distance of 500 mm from 0 < 2θ < 30.2° with a 0.01° step size. SNBL Bubble software was used to integrate the 2D images.^14^ The structural analysis was performed by Rietveld refinements using the JANA2006 software.^13^ For the Rietveld refinement of the synchrotron data, 15 terms of Chebyshev polynomials and the pseudo-Voigt function, applying GU, GW, LX, and LY, were used to fit the background and peak shape, respectively. A Howard (Boole’s rule) parameter was included in the refinement to account for significant peak asymmetry. As the crystal structure had already been solved from single-crystal data, the same structural parameters were used as the starting position for the Rietveld refinement of the powder synchrotron data. During the refinement, the thermal displacement parameters for all oxygen atoms were made identical, and an absorption correction had to be applied because the wavelength applied is close to the L_3_ edge for Sr. Naturally, Berar’s correction was applied to get the correct estimations of standard deviations.^15^

### Neutron Diffraction

A powder sample of SrTe_2_FeO_6_Cl was measured at a high-resolution diffractometer (HRPT) and a cold neutron diffractometer (DMC) at SINQ, Paul Scherrer Institute, Switzerland. The powder was loaded in a 10 mm vanadium can in a He-filled glovebox and sealed with indium. The sample was measured at 60 K at HRPT and at 1.5 and 150 K at DMC, with wavelengths of 1.494 and 2.464 Å, respectively.^16,17^ Rietveld refinements of the diffraction data and the identification of the magnetic symmetry of SrTe_2_FeO_6_Cl were carried out in the JANA2020 software.^13^ A combined refinement of the synchrotron and neutron diffraction data measured at HRPT was used to get a good nuclear model for further refinements of the low-temperature neutron diffraction data measured at DMC. For both data sets, the pseudo-Voigt function was applied; however, for the neutron measurement, GW, GU, GV, and LX were used, while for the synchrotron measurement, GW, GU, LX, and LY were used. To fit the background of the neutron data, 15 Legendre terms combined with a manual background were used, while 15 Chebyshev terms were used for the synchrotron data. In both cases, asymmetry was accounted for using correction by divergence, refining the HpS/L parameter for the neutron data, and using Howard (Boole’s rule) for the synchrotron data. To correct for sample displacement in the neutron beam for the data measured at HRPT, sycos and sysin were used in the refinement. As the two measurements were performed at different temperatures, the X-ray wavelength used in the synchrotron measurement was refined. The standard Berar’s correction was applied to estimate the standard deviations correctly, and all ADP values were made identical.^15^ When refining the low-temperature neutron diffraction data, the background and peak profile were fitted using the same parameters as in the high-temperature neutron data. Once the magnetic unit cell was found, all atomic fractional coordinates were fixed to stabilize the calculations. When left unrestricted, the thermal displacement parameters became negative. To avoid negative thermal displacement parameters, they were fixed toward the end of the refinement. All figures of the magnetic structure were made using the VESTA software.^18^

### Ultraviolet–Visible Spectroscopy

UV–vis reflectance spectra were collected with a Flame-S spectrometer from Ocean Optics using a white diode and optical fibers. The incident light was oriented normal to the sample surface, while the detector was at a 45° angle to this. Absorbance spectra were calculated from reflectance using the Kubelka–Munk approach.^19^

## Results

**Figure 1:**
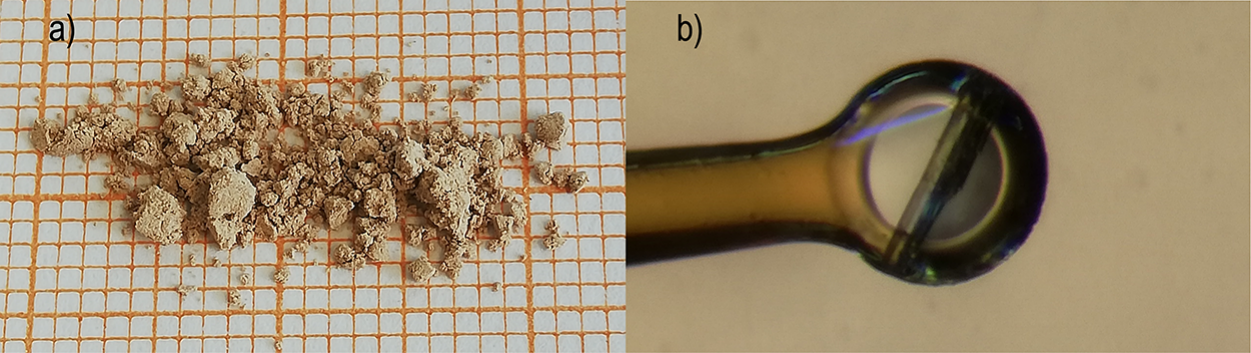
(a) Polycrystalline SrTe_2_FeO_6_Cl sample on top of millimeter paper. (b) SrTe_2_FeO_6_Cl crystal held in place by transparent grease and a sample holder, which measures 75 μm in diameter.

The polycrystalline SrTe_2_FeO_6_Cl powder is light-brown (Figure 1a), which is in accordance with UV–vis data, as the highest absorption occurs at low wavelengths (Figure S1). After syntheses of the polycrystalline SrTe_2_FeO_6_Cl sample, some red powder residues remained on top of the alumina crucible and some small metallic particles were observed on top of the ampule. These observations are believed to be due to small amounts of unreacted Fe_2_O_3_ and metallic Te, respectively. During the EDX analysis, a small crystallite of Te was also observed. A crystal of SrTe_2_FeO_6_Cl is shown in Figure 1b.

**Figure 2:**
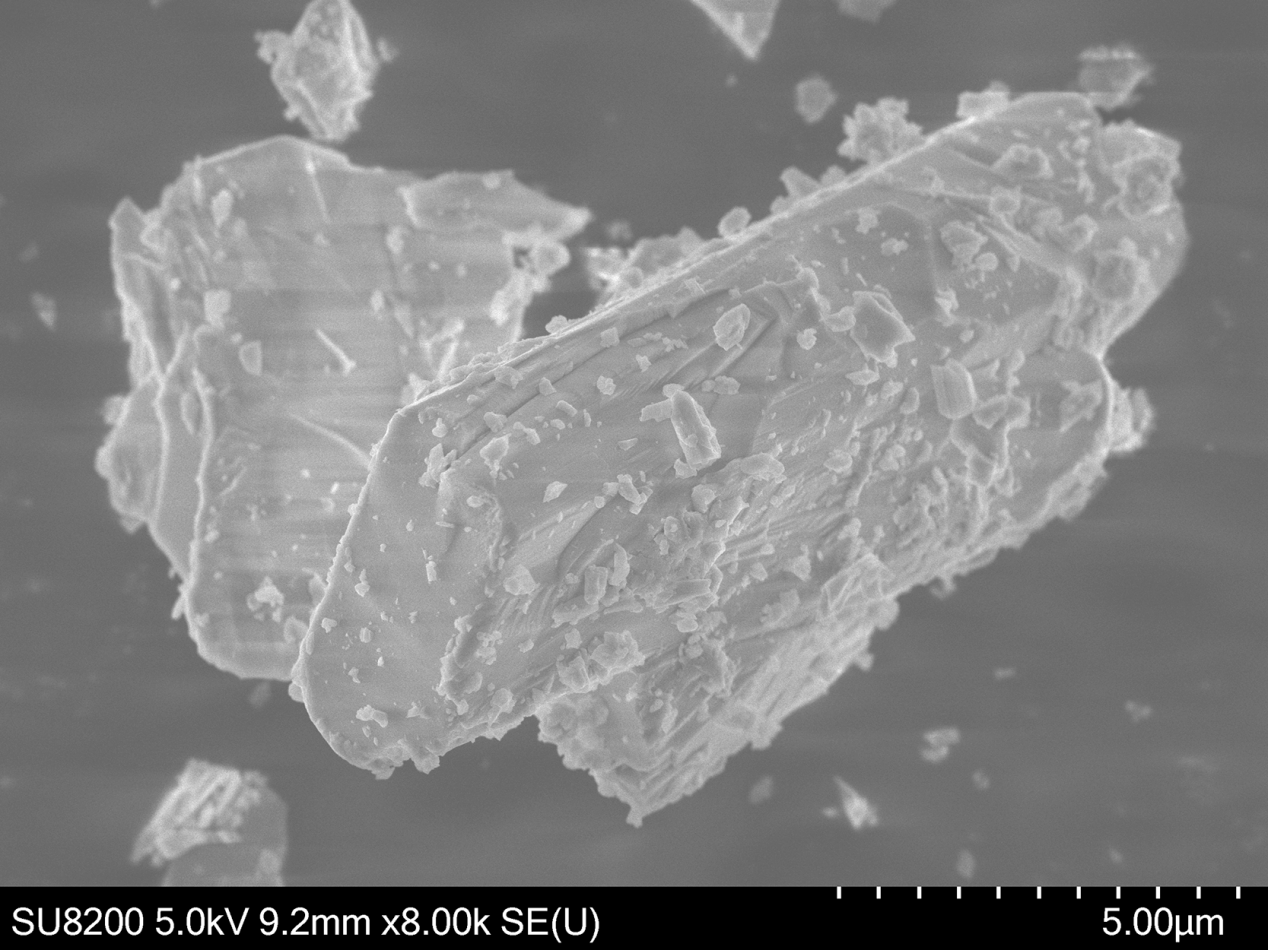
SEM image of multiple crystallites of SrTe_2_FeO_6_Cl. The total length of the added scale bar is 5.00 μm.

The overall elemental composition determined by EDX, assuming six oxygen per formula unit and that +III and +IV are the oxidation states for Fe and Te, respectively, is Sr_1.07(6)_Te_1.99(4)_Fe_0.97(4)_O_6_Cl_0.99(3)_, which is in good agreement with the chemical formula given by the crystal structure. An SEM image of some larger crystallites of SrTe_2_FeO_6_Cl is shown in Figure 2.

### Crystal Structure

**Figure 3:**
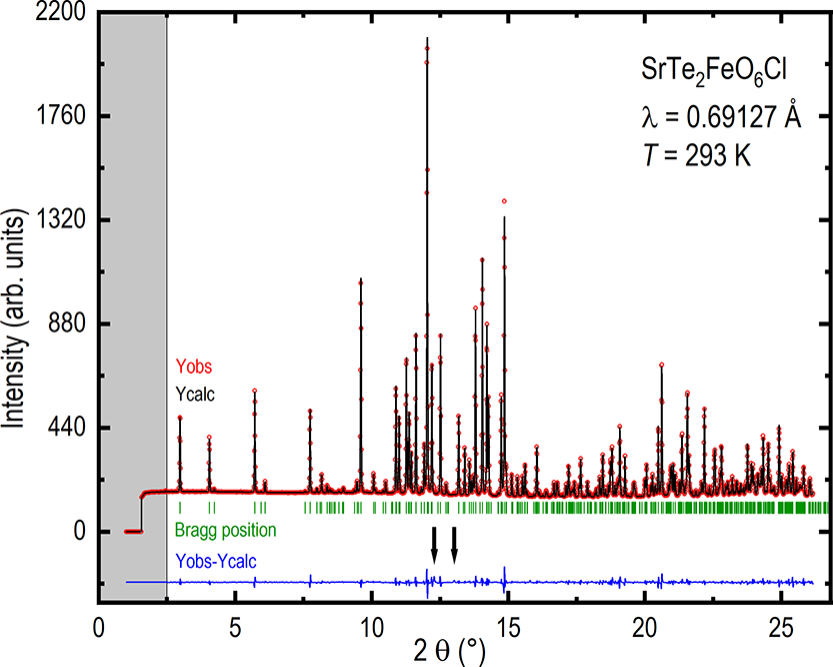
Rietveld refinement of the synchrotron data. *Y*_obs_ (red) is the observed diffractogram, *Y*_calc_ (black) is the calculated diffractogram, Bragg position (green) is the positions of the Bragg peaks, and *Y*_obs_–*Y*_calc_ (blue) is the difference between observation and calculation. The area shown in gray was not included in the refinement. The two black arrows indicate where the strongest reflections from γ-TeO_2_ would be expected.^20^

The crystal structure of SrTe_2_FeO_6_Cl was determined using XRD. Rietveld refinement of the synchrotron diffraction data is shown in Figure 3. Except for a few tiny diffraction intensities, all intensities agree with the primary phase. In the figure, two black arrows indicate where the two strongest reflections from γ-TeO_2_ would be expected, matching well with the strongest extrinsic reflections.^20^ A similar refinement of the pXRD data is shown in Figure S2.

As the Rietveld refinement of the synchrotron data led to a better agreement between observation and calculation and, combined with the fact that SrTe_2_FeO_6_Cl crystallizes in a centrosymmetric space group, the obtained structure parameters from the Rietveld refinement of the synchrotron data are summarized in Table 1. The structure parameters obtained by single-crystal XRD are shown in Table S1, and the Rietveld refined neutron diffraction data measured at 60 K at HRPT, as part of the combined refinement of neutron and synchrotron data, later used to determine the magnetic structure, are shown in Figure S3.

**Table 1:** Results from the Rietveld Refinement of the Synchrotron Data for SrTe_2_FeO_6_Cl

chemical formula	SrTe_2_FeO_6_Cl
fw (g mol^–1^)	530.112
temperature	ambient
λ (Å)	0.69127
crystal system	monoclinic
space group	*P*12_1_/*n*1 (no. 14)
*a* (Å)	10.2604(1)
*b* (Å)	5.34556(5)
*c* (Å)	26.6851(3)
*V* (Å^3^)	1460.58(2)
β (°)	93.6853(4)
*Z*	8
GOF	0.26
*R*_P_ (%)	1.32
*R*_Wp_ (%)	2.92
diff Fourier peak/hole (e Å^–3^)	0.19/–0.18
CSD number	2266330

**Figure 4:**
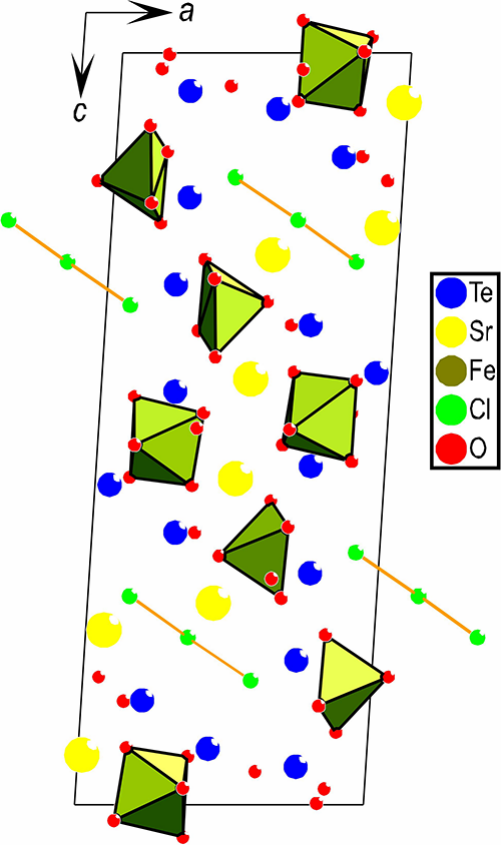
Unit cell for SrTe_2_FeO_6_Cl with all chlorines connected and the different iron coordinations displayed.

The unit cell for SrTe_2_FeO_6_Cl is shown in Figure 4, with all chlorines connected and the different iron coordinations displayed.

**Figure 5:**
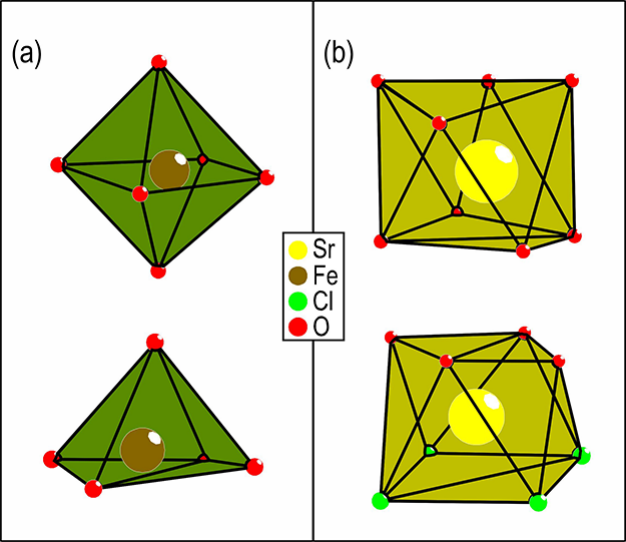
Different coordinations observed for iron (a) and strontium (b) in SrTe_2_FeO_6_Cl.

**Figure 6:**
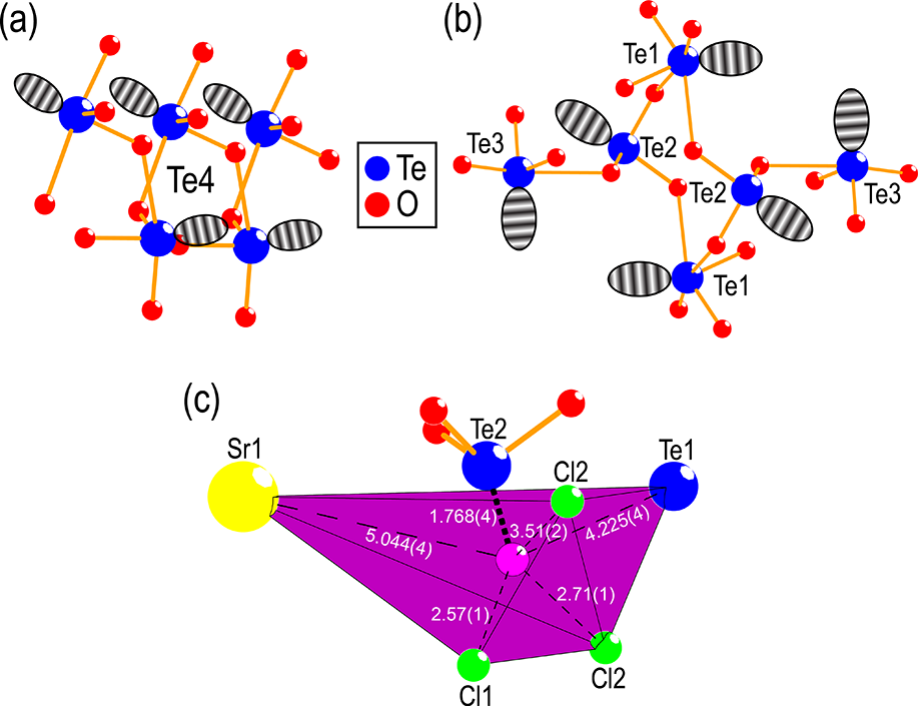
Different coordinations observed for tellurium in SrTe_2_FeO_6_Cl. (a) One-dimensional chain of trigonal bipyramidal [TeO_4_*E*] units. (b) [Te_6_O_18_]^12–^ unit made up of Te1, Te2, and Te3. The lone pair for Te(IV) is shown using faded eclipses placed by the hand. (c) Closest surrounding of the assumed lone pair volume for Te2, with the purple sphere indicating the center of the coordination. All distances are given in Å.

Two different homoleptic coordinations, spatially separated in the atomic lattice, are observed for Fe, one distorted octahedron and one distorted square pyramid, as shown in Figure 5a. The Fe–O interatomic distances vary between 1.92–2.12 and 1.86–2.08 Å for the octahedral and square pyramidal coordination, respectively. This is comparable to the Fe–O distances observed in the octahedral coordination in Fe_2_O_3_, which varies between 1.946 and 2.116 Å.^21^ The closest Fe–Cl distance is 3.13 Å, which is significantly longer than the 2.378 Å Fe–Cl distance observed in FeCl_3_^22^ and is therefore not considered part of the coordination sphere of the square pyramidal coordinated iron. The fractional atomic coordinates for all atoms and the bonding distances for the different coordination polyhedra shown in Figures 5 and 6 are given in Tables S2 and S3 in the Supporting Information, respectively.

In the case of Sr, two different coordinations are observed. One Sr is homoleptic coordinated to eight oxygen atoms, and the other Sr is heteroleptic coordinated to four chlorine and four oxygen in a *fac*-like manner, as shown in Figure 5b.

Four different Te atoms are found within the structure, all taking the classical distorted tetrahedral [TeO_3_*E*] coordination, where *E* is the lone pair, as expected for Te(IV). The Te–O distances vary between 1.81 and 1.96 Å, similar to those found in related structures such as Co_2_TeO_3_Cl_2_^4^ and Cu_2_Te_2_O_5_Cl_2_.^3^ However, three of the four Te atoms have either one or two additional oxygen atoms within 2.65 Å, which should be considered part of the coordination according to bond valence sum (BVS) calculations. For Te4, this results in a 1D chain of trigonal bipyramidal [TeO_4_*E*] units, as shown in Figure 6a. Te1, Te2, and Te3 form a ring-structured [Te_6_O_18_]^12–^ unit (Figure 6b) where Te1 coordinates to five oxygen forming a distorted octahedron, [TeO_5_*E*]. Te2 only has three oxygen within 2.65 Å and remains in a distorted tetrahedral coordination. Similar to Te4, Te3 forms a trigonal bipyramidal coordination [TeO_4_*E*]. However, the BVS calculations might be slightly inhibited by the lone pair of Te(IV). The lone pair coordination of Te2 is shown in Figure 6c, highlighting the closest surrounding of the assumed lone pair volume, with the center of the coordination being indicated by the purple sphere.

**Figure 7:**
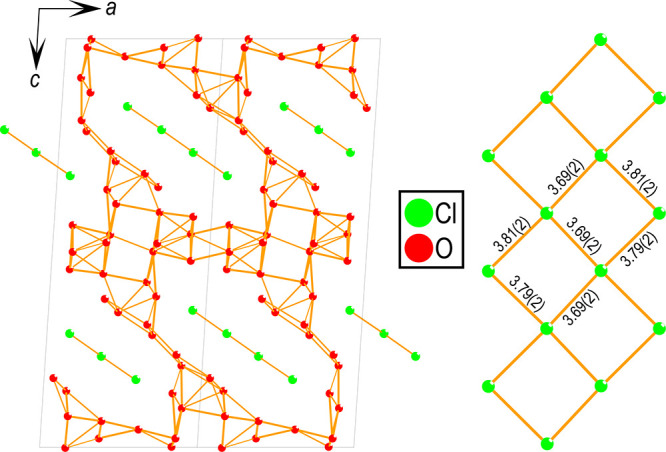
Anion ordering present in SrTe_2_FeO_6_Cl. To the left: two unit cells showing how the chlorine (green) structures are surrounded by an oxygen (red) matrix. To the right: a closer look at one of the chlorine zig-zag ladders with distances shown in Å. Cations are omitted for clarity.

An interesting anion ordering is observed, as shown in Figure 7, with the chlorine atoms forming a zig-zag ladder of almost perfect squares of chlorine, separated by an irregular oxygen matrix. Interestingly, the lone pair of tellurium (not shown) is oriented toward the chlorine zig-zag ladders, as described previously in the literature.^10^ Different types of chlorine lattices have previously been observed in oxychlorides, such as Bi_4_TaO_8_Cl^23^ (2D layers) and La_3_WO_6_Cl_3_^24^ (1D tunnels). To the best of the authors’ knowledge, this is the first observation of this particular anion ordering of chlorine forming zig-zag ladders.

### Magnetic and Specific Heat Measurements

**Figure 8:**
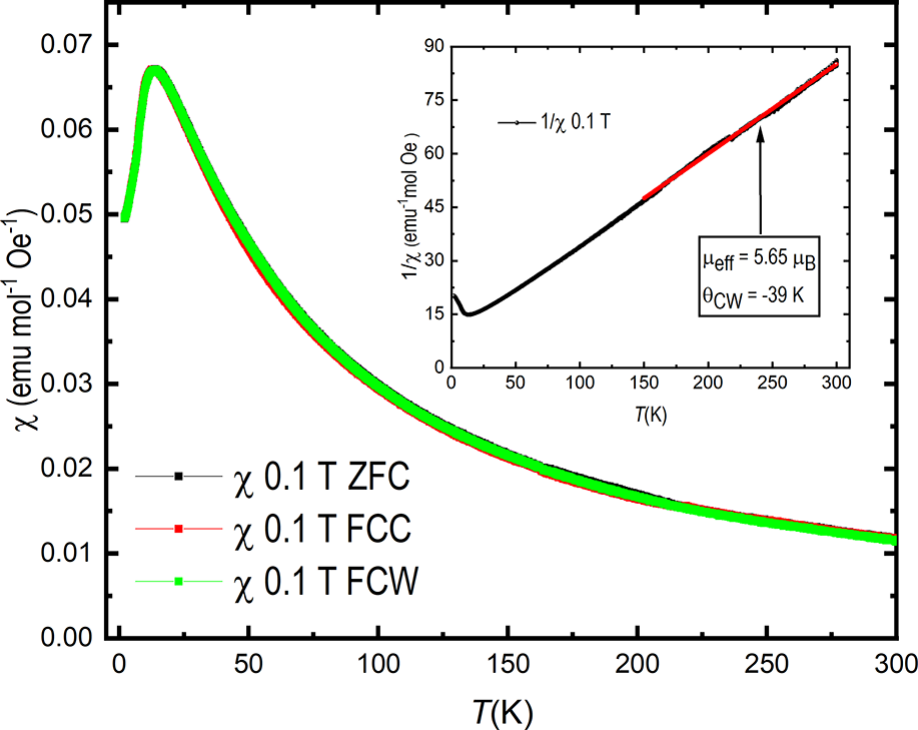
Magnetic susceptibility for SrTe_2_FeO_6_Cl as a function of temperature as measured in both zero-field cooled (ZFC), field cooled cooling (FCC), and field cooled warming (FCW) modes. Displayed in the inset is the inverse magnetic susceptibility plot for SrTe_2_FeO_6_Cl. The linear Curie–Weiss regression curve is shown in red.

Magnetic susceptibility measurements suggest that the title compound exhibits typical antiferromagnetic (AFM) behavior in its ground state (Figure 8). The maximum susceptibility is observed at Néel temperature, *T*_N_ ∼ 13 K.

The high-temperature range of the inverse magnetic susceptibility (inset Figure 8) can be fitted to a Curie–Weiss approximation with satisfactory agreement. From the linear fit, an effective paramagnetic magnetic moment of μ_eff_ = 5.65 μ_B_ Fe^–1^ is determined, close to the expected value for a high-spin *d*^5^ cation (μ_eff_ =  μ_B_). The corresponding Weiss constant, θ_CW_ = −39 K, also agrees with AFM ordering at low temperatures. From a *M* vs *H* measurement performed at 300 K, no indication of any ferromagnetic impurities could be observed, as shown in Figure S4.

**Figure 9:**
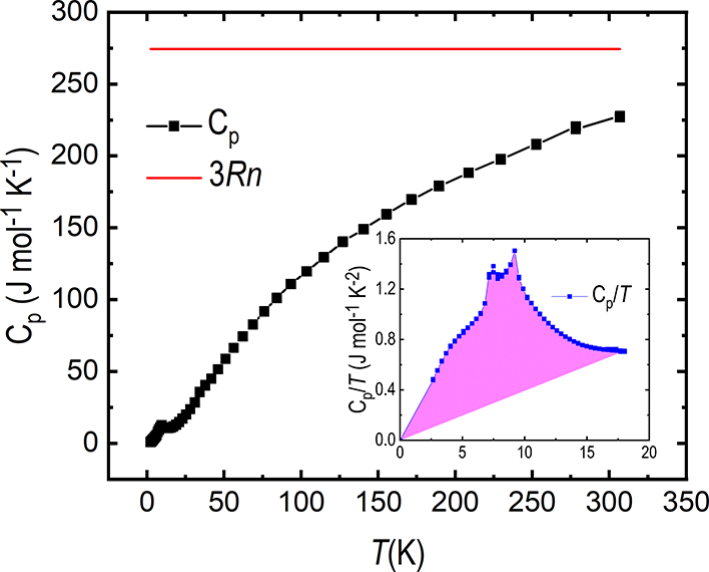
Specific heat capacity measurement for SrTe_2_FeO_6_Cl (black) and the calculated Dulong–Petit limit (red, *n* = 11). The blue curve in the inset highlights the low-temperature part of the specific heat capacity measurement for a higher resolution measurement scaled to match the black curve at high temperatures. The pink area is a rough estimate of the release of heat due to magnetic ordering and corresponds to 8.5 J mol^–1^ K^–1^.

Data from the specific heat capacity measurement, performed on a cold-pressed pellet, are shown in Figure 9. *C*_p_/*T* from a higher resolution measurement is shown in the inset, and the data were scaled to match the black curve for high temperatures. Two anomalies are discernable between 6 and 9 K in the inset, corresponding to the release of heat, likely due to magnetic ordering. The coupling between the sample and the sample holder was >90% during the measurement. The Dulong–Petit limit, shown in red, is not reached at room temperature, but the discrepancy is not alarming.

To get a better approximation of the heat release at low temperatures, the point (0,0) was added to the data set and the area under the curve, shown in the inset in Figure 9, was roughly estimated. This estimation of the phonon background should serve as a lower limit for its contribution. The resulting area is shown in pink and corresponds to 8.5 J mol^–1^ K^–1^, significantly lower than the expected 14.9 J mol^–1^ K^–1^ (R*ln(2*S* + 1)) for full long-range AFM ordering. However, without a nonmagnetic reference, it is difficult to get a true estimate of the phonon background.

### Magnetic Structure

**Figure 10:**
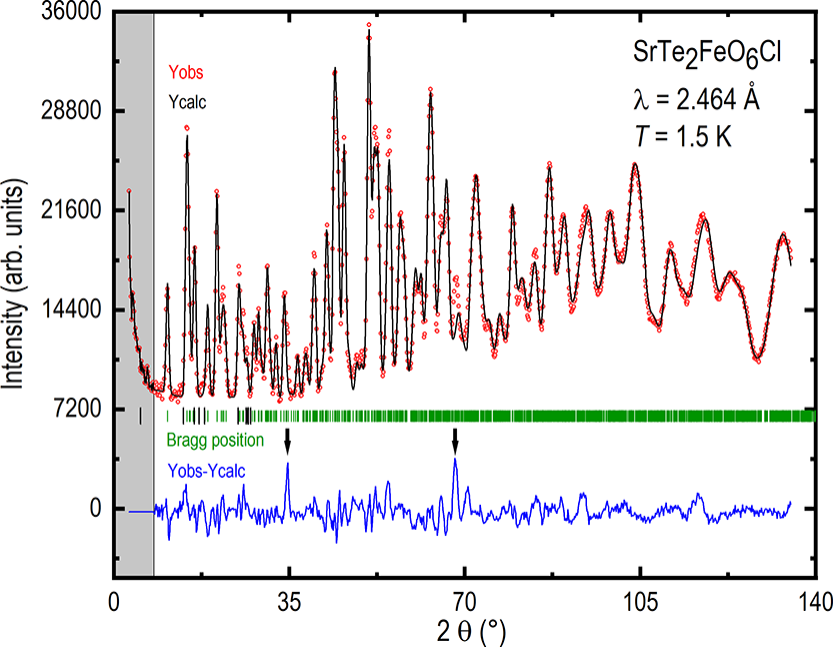
Rietveld refinement of the neutron diffraction data for SrTe_2_FeO_6_Cl collected at 1.5 K. *Y*_obs_ (red) is the observed diffractogram, *Y*_calc_ (black) is the calculated diffractogram, green vertical lines indicate nuclear Bragg scattering, while thick black lines indicate the first 10 contributions from magnetic scattering, and *Y*_obs_–*Y*_calc_ (blue) is the difference between the observed and calculated diffractogram. The area in gray was not included in the refinement. The use of black arrows highlights the two largest peaks originating from an unknown phase.

The low-temperature neutron diffraction measurements revealed the presence of additional peaks (Figure S5), which could be indexed using a propagation vector (1/2, 1/2, 0) found using the WinPLOTR-2006^25,26^ software. When trying to solve the magnetic ordering, it was directly apparent that the magnetic moment along the *x*- and *y*-directions stayed very close to zero and was hence restricted to zero. Two different spin structures gave an equally good fit to the data, making it impossible to distinguish the different magnetic orderings. The Rietveld refinement of the magnetic structure is shown in Figure 10. The two most prominent peaks, originating from an unknown phase, are highlighted using black arrows. Comparing the neutron data collected at 1.5 and 150 K (Figure S5), above and below the ordering temperature of the material, it is evident that the same two unidentified peaks are present and are not caused by the magnetic ordering of the sample.

**Figure 11:**
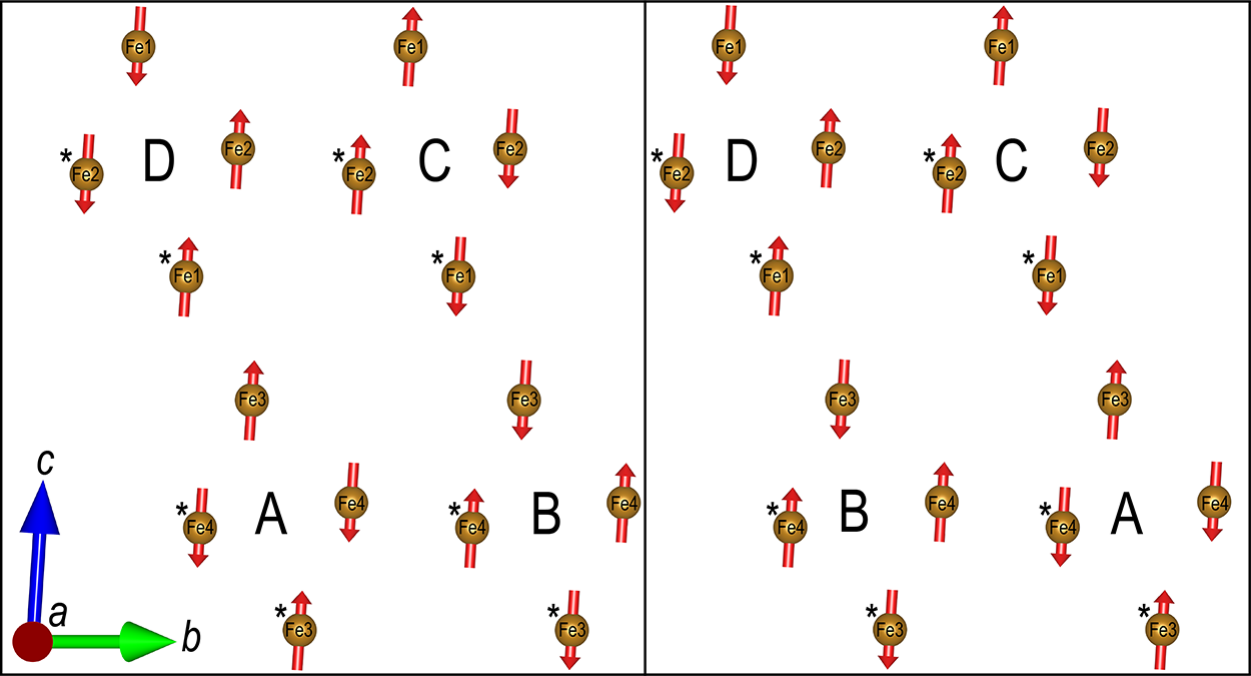
Two indistinguishable magnetic structures determined for SrTe_2_FeO_6_Cl viewed along the *a*-axis. Only two layers of iron atoms along the *a*-axis are shown for clarity. Marked and unmarked Fe atoms belong to the top and bottom layer along the *a*-axis, respectively.

The two indistinguishable spin structures, viewed along the *a*-direction, are shown in Figure 11. Here, only two layers of Fe atoms along the *a*-direction are shown. Fe atoms belonging to the top layer are marked with an asterisk (*), and Fe atoms belonging to the bottom layer are unmarked, emphasizing the antiferromagnetic layers, which repeat in the *a*-direction. If more layers along the *a*-direction are included, they are stacked directly above previous atoms with their magnetic moment in the opposite direction. The Fe atoms form layers of squares (layers of rows of squares if additional layers of Fe along the *a*-axis are included) that differ in magnetic ordering, shown using A, B, C, or D in Figure 11. The two layers either order in an A–B or D–C fashion and alternate along the *c*-direction. Comparing the two different structures, it is clear that they are related by a shift along one of the two layers.

The resulting structural parameters from the Rietveld refinement of the low-temperature neutron diffraction data are summarized in Table 2.

**Table 2:** Structure Parameters Determined by Rietveld Refinement of the Low-Temperature Neutron Diffraction Data Determined for SrTe_2_FeO_6_Cl

chemical formula	SrTe_2_FeO_6_Cl
temperature (K)	1.5
λ (Å)	2.464
crystal system	triclinic
magnetic space group	*P*–1.1′*c*[*P*–1]
*a* (Å)	10.6693
*b* (Å)	11.5422
*c* (Å)	26.6172
*V* (Å^3^)	2900.674
α (°)	86.7241
β (°)	90
γ (°)	62.4718
*Ζ*	16
GOF	5.0499
*R*_P_ (%)	1.60
*R*_Wp_ (%)	2.29
diff Fourier peak/hole (e Å^–3^)	0.12/–0.14
Fe1 moment along |*z|* (μ_B_)	4.14(7)
Fe2 moment along |*z|* (μ_B_)	4.14(7)
Fe3 moment along |*z|* (μ_B_)	4.14(7)
Fe4 moment along |*z|* (μ_B_)	4.14(7)

## Discussion

Elemental composition determined by EDX and electron densities from SC-XRD data support that SrTe_2_FeO_6_Cl is the correct chemical composition. This is further confirmed by the fact that an almost X-ray pure sample could be synthesized when using this starting composition for a high-temperature reaction. However, there are some issues with Te(IV) being reduced at higher temperatures, as evidenced by observing metallic particles inside an ampule and the Te metal during the EDX analysis.

When refining the neutron data, two different magnetic orderings could not be distinguished during the refinement. Further magnetic studies are required to determine the magnetic structure; for instance, single-crystal neutron diffraction might be sufficient to determine the magnetic structure unambiguously. A lower magnetic moment of 4.14 μ_B_ is observed compared to the expected value of 5 μ_B_ for Fe^3+^, a *d*^5^ cation, as is often observed for AFM-ordered samples.^27^

**Figure 12:**
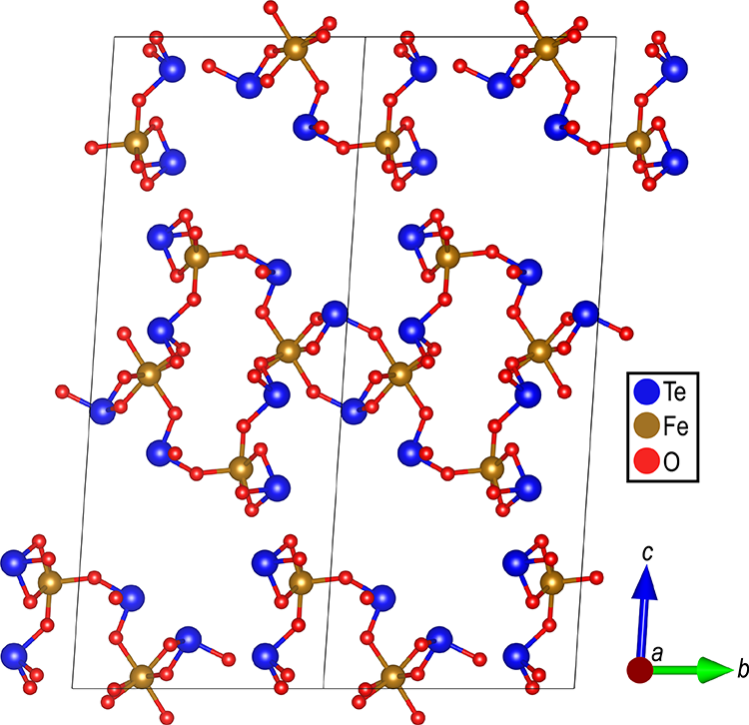
Magnetic unit cell for SrTe_2_FeO_6_Cl, shown using two unit cells along the *b*-axis. Only tellurium, iron, and oxygen atoms are included, and the magnetic moment for iron is omitted for clarity.

From the susceptibility and neutron diffraction measurements, it is clear that the sample shows long-range AFM ordering at low temperatures. As shown in the inset in Figure 9, two distinct peaks are present in the high-resolution specific heat capacity measurement at low temperatures, likely due to the release of heat at the magnetic ordering. A more detailed inspection of the magnetic structure reveals that no classical superexchange (Fe–O–Fe) is present, meaning that the magnetic exchange is likely between iron, oxygen, and tellurium (Fe–O–Te–O–Fe). If only Te–O and Fe–O interatomic distances shorter than 2.55 and 3.38 Å, respectively, are considered, no bonding is observed between the layers of squares formed by the Fe atoms; however, within each layer, multiple Fe–O–Te–O–Fe coordinations exist, as shown in Figure 12.

**Figure 13:**
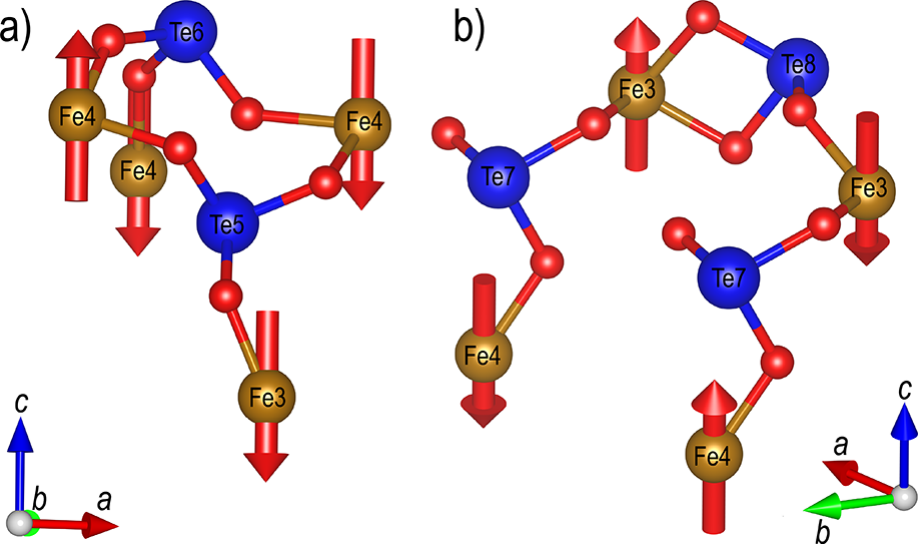
Different Fe–O–Te–O–Fe coordination observed for SrTe_2_FeO_6_Cl. (a) Te5 and Te6 coordinated to three iron atoms. (b) Te7 and Te8 coordinated to two iron atoms. All atoms not involved in the Fe–O–Te–O–Fe coordination have been removed for clarity.

Inspection of each Te atom reveals that all are coordinated to two or three iron atoms, as shown in Figure 13. Te5 and Te6 are coordinated to three iron atoms (Figure 5a), while Te7 and Te8 are coordinated to two iron atoms (Figure 5b); Te8 is also doubly coordinated to one iron atom. The existence of multiple exchange paths of different coupling strengths is a tentative explanation for the two peaks observed in the specific heat measurement, in agreement with a previous report.^28^

As previously mentioned, combining p-block elements with stereochemically active lone pairs and halide ions often leads to low dimensionality. This can be explained by the fact that the lone pair is often directed toward the halide ions, creating large areas without bonding.^10^ Comparing related compounds, in both Co_2_TeO_3_Cl_2_ and SrCo_2_Te_3_O_8_Cl_2_, the same observation is made; the lone pair of Te(IV) is directed toward the chlorine atoms. This observation can be rationalized using the anion charges (Cl^–^ and O^2–^); the lone pair will be directed toward the least negative anion to reduce repulsion between the lone pair and the negative anions. Comparing the shortest Te–Cl distances observed for Co_2_TeO_3_Cl_2_, SrCo_2_Te_3_O_8_Cl_2_ and the title compound yield 3.054,^4^ 3.05,^10^ and 3.13 Å, respectively, all very similar values. Using p-block elements with stereochemically active lone pairs and halide ions is a possible chemical approach to synthesizing novel low-dimensional crystal structures.

## Conclusions

A new compound, SrTe_2_FeO_6_Cl, has been discovered, which crystallizes in a novel monoclinic crystal structure with unit cell parameters, *a* = 10.2604(1) Å, *b* = 5.34556(5) Å, *c* = 26.6851(3) Å, and β = 93.6853(4)°, as indicated by synchrotron diffraction and EDX. Magnetic investigations show that the sample is a C-type antiferromagnet with a Néel temperature of 13 K, and the magnetic and nuclear unit cells are related by a propagation vector (1/2, 1/2, 0). The specific heat capacity measurement further supports long-range antiferromagnetic ordering, which shows the release of heat close to the expected ordering temperature. This work highlights an already established way of chemically designing structures with low dimensionality by incorporating elements with stereochemically active lone pairs, halide ions, and large alkaline-earth metals. Further investigations into similar compositions are a promising route for discovering new low-dimensional crystal structures.

## Supporting Information Available

UV–vis results, Rietveld refinement of pXRD and neutron data, comparison of neutron data measured at different temperatures, SC-XRD data, *M* vs *H* measurement, and fractional atomic positions and bonding distances (PDF)
