# Supplementary material for: X-ray and Neutron Diffraction Studies of SrTe2FeO6Cl, an Oxide Chloride with Rare Anion Ordering
Source: Inorg Chem. 2023 Aug 2;62(32):13081–8. doi: 10.1021/acs.inorgchem.3c01951 (PMC10428212; doi:10.1021/acs.inorgchem.3c01951)
Supplement: Supplementary file 1 — ic3c01951_si_001.pdf [file ic3c01951_si_001.pdf]

Supporting Information

X-ray and Neutron Diffraction Studies of $SrTe_2FeO_6Cl$, an Oxide Chloride with Rare Anion Ordering.

Johnny A. Sannes,[1] Bruno Gonano,[1] Øystein S. Fjellvåg,[2,3] Susmit Kumar,[1,4] Ola Nilsen,[1] Martin Valldor[1]*

[1] Centre for Materials Science and Nanotechnology (SMN), Department of Chemistry, University of Oslo, Sem Sælands vei 26, N-0371 Oslo, Norway

[2] Department for Hydrogen Technology, Institute for Energy Technology, NO-2027 Kjeller, Norway

[3] Laboratory for Neutron Scattering and Imaging, Paul Scherrer Institute, Forschungsstrasse 111, Villigen PSI, 5232, Switzerland

Corresponding Author

*Johnny Sannes, email: j.a.sannes@kjemi.uio.no

To further support the presence of Fe^{3+} in the sample, pellets of Fe_2O_3 (Fluka, >97%), $FeSO_4*7H_2O$ (Fluka, p.a) and the title compound were pressed, and UV-VIS reflection measurements were performed and recalculated to equivalent absorption spectra using the Kubelka-Munk approach[1]. The comparison of the spectra measured for the sample and the two references is shown in figure S1.

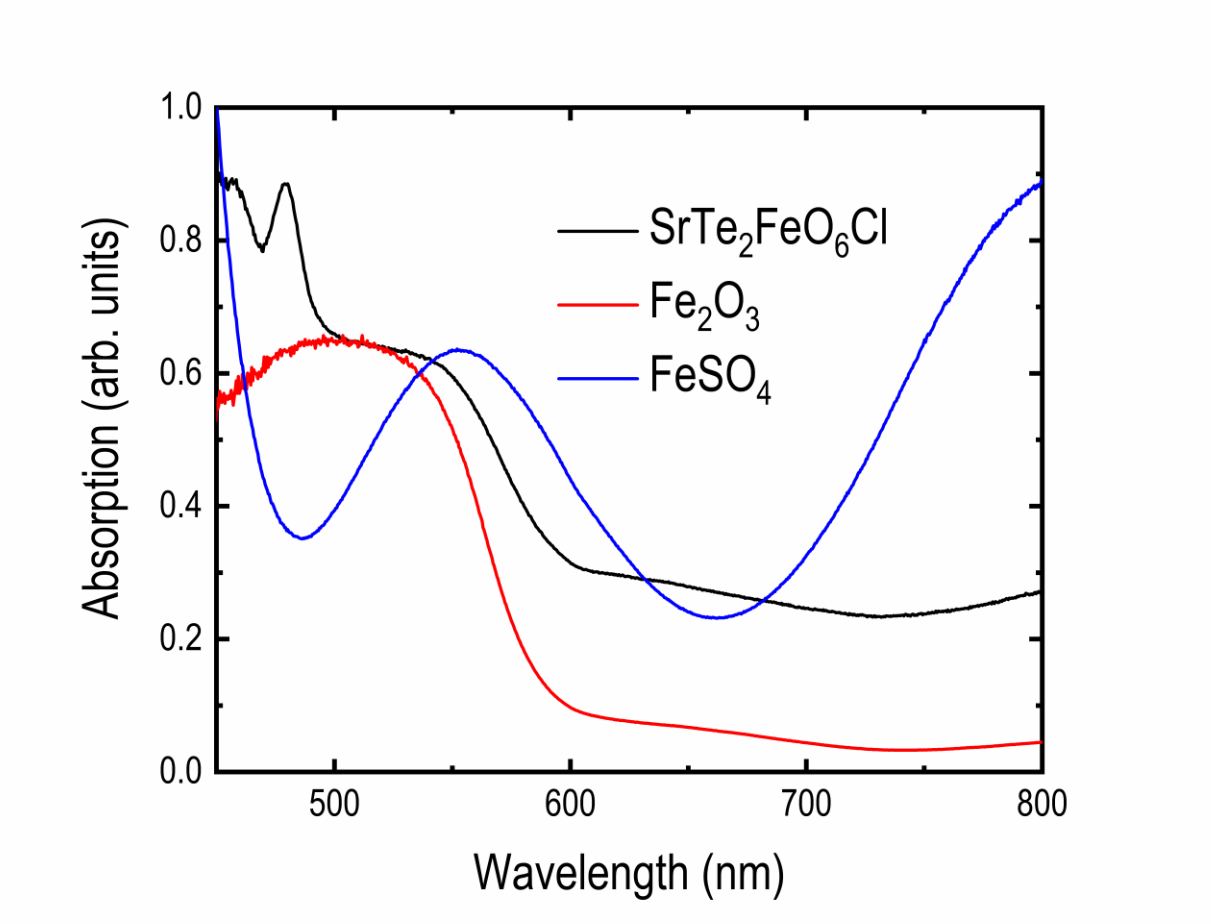

Figure S1. Comparison of the UV-VIS spectra for $SrTe_2FeO_6Cl$ (black) and the two references, Fe_2O_3 (red) and $FeSO_4$ (blue).

The UV-VIS data measured for Fe_2O_3 and $FeSO_4$ was scaled to roughly match the measured data for $SrTe_2FeO_6Cl$ at 550 nm. Comparing the different graphs, it is evident that if Fe^{2+} were the primary constituent, notable absorption at 450 nm and particularly at 800 nm would be expected, while this is not observed. The curve observed for Fe^{3+} (Fe_2O_3) is significantly more representative of the sample.

In order to evaluate the purity of the sample, a Rietveld refinement of the pXRD data was performed, and the result is shown in figure S2. The refinement was performed almost identically to the synchrotron refinement; however, all ADP parameters were made identical, Berar's correction was not applied, and the structural parameters from the refinement of the synchrotron data were used as the basis for the Rietveld refinement. The refinement was significantly improved when the March-Dollase function was included to simulate crystalline texturing, i.e. preferred orientation along (0 1 0). A few intensities from unidentified secondary phases are observed, with the strongest reflection close to 2θ = 29.4°. The identity of this impurity is unclear; however, the two black arrows indicate where the strongest reflections from γ-TeO_2 would be expected[2], which matches well with the difference curve in figure S2.

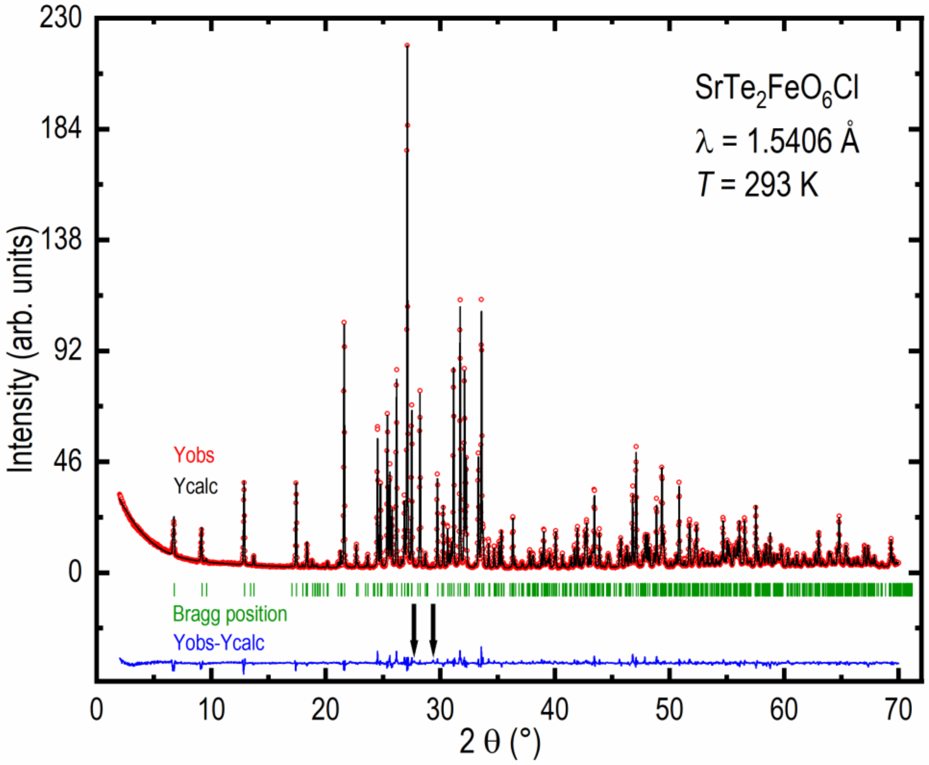

Figure S2. Rietveld refinement of the pXRD data for $SrTe_2FeO_6Cl$. Yobs (red) is the observed diffractogram, Ycalc (black) is the calculated diffractogram, Bragg position (green) is the positions of the Bragg peaks, and Yobs-Ycalc (blue) is the difference between observation and calculation. The two black arrows indicate where the strongest reflections from γ-TeO_2 would be expected[2].

The Rietveld refined neutron data measured at 60 K, as part of the combined refinement of neutron and synchrotron data, is shown in figure S3. The position of the two black arrows corresponds to the position of the two peaks in the DMC data (figure 10) when accounting for the different wavelengths used.

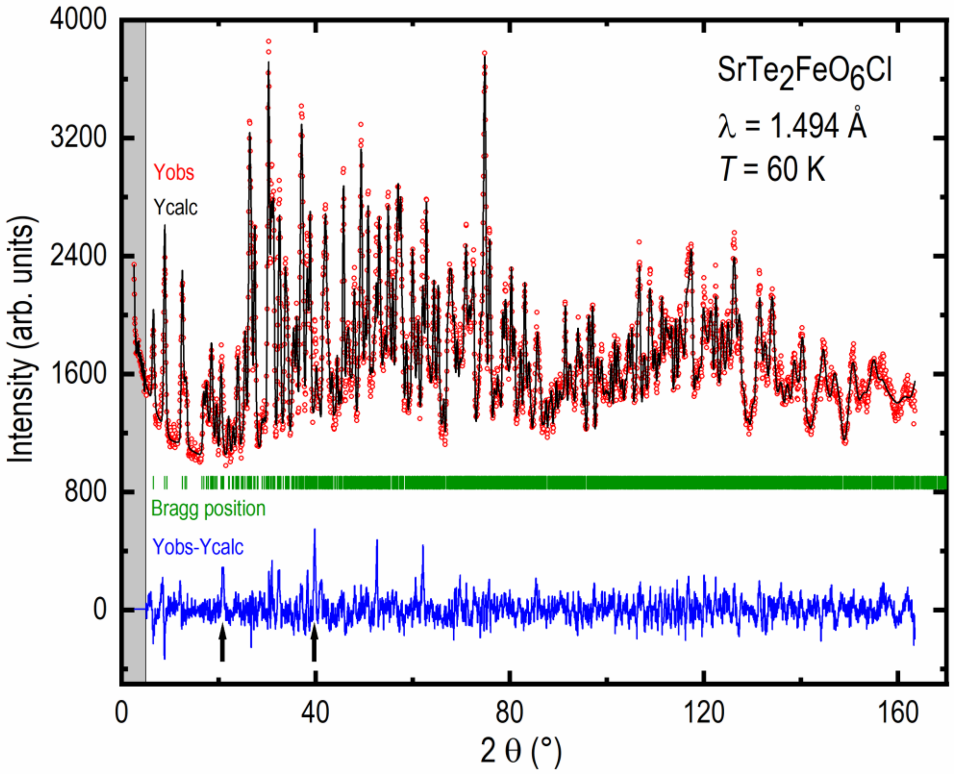

Figure S3. Rietveld refinement of the neutron diffraction data for $SrTe_2FeO_6Cl$ collected at 60 K. Yobs (red) is the observed diffractogram, Ycalc (black) is the calculated diffractogram, Bragg position (green) is the positions of the Bragg peaks, and Yobs-Ycalc (blue) is the difference between the observed and calculated diffractogram. The area in grey was not included in the refinement. The black arrows indicate the 2θ values of the two peaks observed in the low-temperature neutron data if λ = 1.494 Å was used.

To investigate the possibility of any ferromagnetic impurities, a *M* vs *H* measurement at 300 K was performed, as shown in figure S4. From the measurement, no signs of any ferromagnetic impurities could be observed.

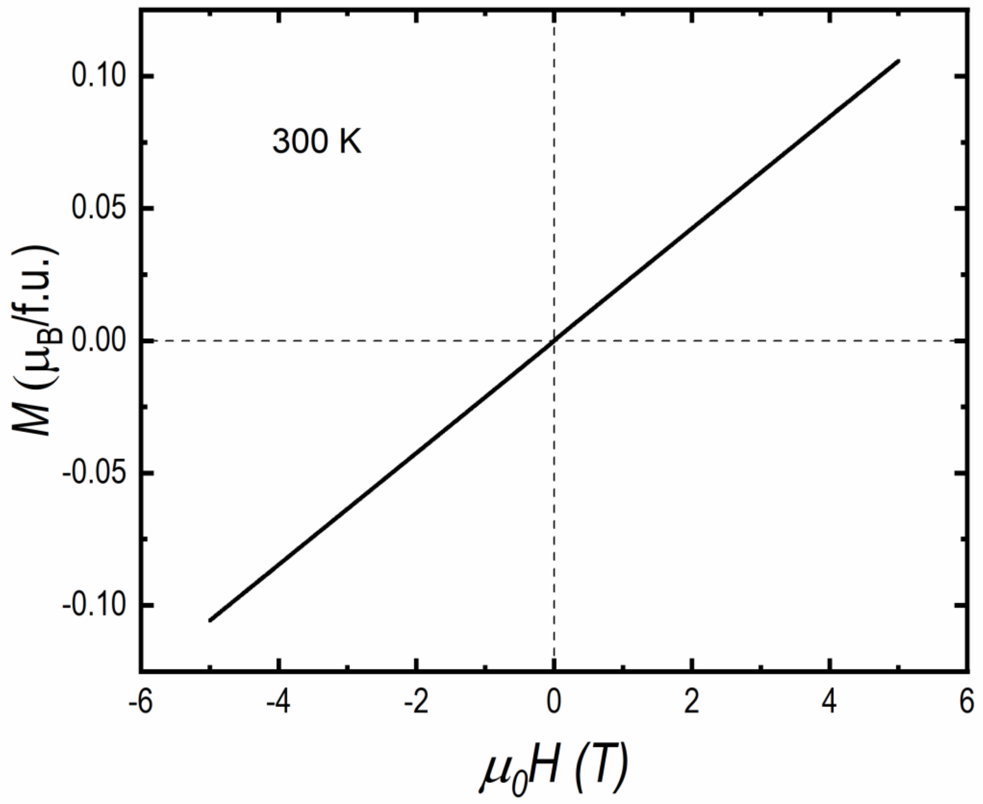

Figure S4. *M* vs *H* measurement performed at 300 K for $SrTe_2FeO_6Cl$.

A comparison between the neutron diffraction data measured at 1.5 K and 150 K is shown in figure S5. Comparing the two measurements, one can identify the magnetic reflections. From the Rietveld refinement of the neutron data measured at 1.5 K, the magnetic unit cell could not explain two peaks, as shown in figure 10. The same peaks are marked by black arrows in figure S5. As the two neutron diffraction measurements were performed above and below the ordering temperature of the title compound, these peaks are, therefore, from an unidentified secondary phase.

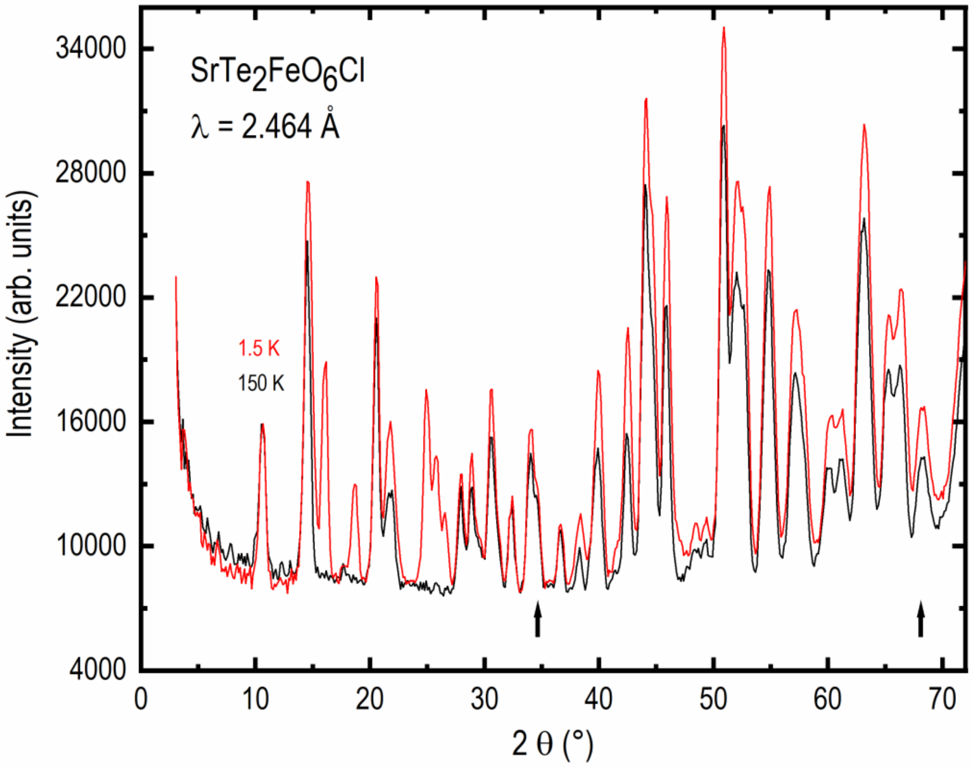

Figure S5. Comparison of the neutron diffraction data collected at 1.5 K and 150 K. The black arrows indicate the position of the two peaks that could not be explained by the magnetic unit cell during the Rietveld refinement of the neutron data collected at 1.5 K.

The obtained structure parameters from single-crystal XRD data are shown in table S1.

Table S1. The obtained structure parameters from single-crystal XRD data.

chemical formula	$SrTe_2FeO_6Cl$
fw (g mol^{-1})	530.112
temperature	ambient
λ (Å)	0.71073
crystal system	monoclinic
space group	$P12_1/n$ 1 (No.14)
a (Å)	10.250(4)
b (Å)	5.337(2)
c (Å)	26.66(1)
V (Å^3)	1456(1)
β (°)	93.69(1)
Z	8
GOF_{all}/GOF_{obs}	1.67/1.53
R_{all}/R_{obs} (%)	14.51/4.99
$R_{W,all}/R_{w,obs}$ (%)	13.17/8.11
diff Fourier peak/hole (e Å^{-3})	3.79/-4.34
Index ranges	$-13 \leq h \leq 13$; $-7 \leq k \leq 7$; $-36 \leq l \leq 36$

Atomic positions and thermal displacement parameters

The fractional atomic positions and thermal displacement parameters, as determined by Rietveld refinement of the synchrotron data, are shown in table S2.

Table S2. The fractional atomic positions and thermal displacement parameters for $SrTe_2FeO_6Cl$ as determined by Rietveld refinement of the synchrotron data.

Atom	x	y	z	U (Å^2)
Te1	0.2421(3)	0.267(1)	0.05027(9)	0.030(1)
Te2	0.5515(2)	-0.266(1)	0.0745(1)	0.029(2)
Te3	-0.2118(3)	-0.728(1)	0.1386(1)	0.032(1)
Te4	0.2634(3)	0.772(1)	0.1923(1)	0.030(1)
Sr1	-0.0157(4)	-0.248(2)	0.0662(2)	0.032(2)
Sr2	-0.0640(4)	-0.222(2)	0.2323(1)	0.036(2)
Fe1	0.1138(5)	0.224(2)	0.1620 (2)	0.030(2)
Fe2	0.2759(5)	-0.219(2)	-0.0164(2)	0.028(2)
Cl1	-0.355(1)	-0.213(3)	0.2223(4)	0.042(4)
Cl2	0.4171(9)	0.290(3)	0.1652(3)	0.036(5)
O1	0.133(3)	0.530(5)	0.200(1)	0.020(3)
O2	0.372(2)	0.111(4)	-0.0220(9)	0.020(3)
O3	0.166(2)	-0.160(5)	-0.0760(7)	0.020(3)
O4	-0.146(2)	-0.414(5)	0.1378(9)	0.020(3)
O5	0.382(2)	-0.328(5)	0.0437(8)	0.020(3)
O6	0.140(3)	0.000(5)	0.021 (1)	0.020(3)
O7	0.381(2)	-0.403(4)	-0.0644(9)	0.020(3)
O8	0.162(3)	0.504(5)	0.0016(9)	0.020(3)
O9	0.169(3)	0.015(4)	0.2256(9)	0.020(3)
O10	0.178(2)	-0.084(4)	0.1313(8)	0.020(3)
O11	0.113(2)	0.367(4)	0.0960(9)	0.020(3)
O12	-0.057(2)	0.124(4)	0.1695(8)	0.020(3)

The cation-anion bonding distances for the different coordination polyhedra shown in figures 5 and 6 are summarized in table S3.

Table S3. Summarized cation-anion bonding distances for the different coordination polyhedra shown in figures 5 and 6.

Atom 1	Atom 2	Distance (Å)	Atom 1	Atom 2	Distance (Å)	Atom 1	Atom 2	Distance (Å)
Sr1	O6	2.45(3)	Te1	O6	1.90(3)	Fe1	O12	1.86(2)
	O11	2.55(2)		O11	1.93(2)		O11	1.92(2)
	O4	2.56(2)		O8	1.96(3)		O1	1.92(3)
	O8	2.65(3)		O2	2.55(2)		O10	1.97(3)
	O3	2.69(3)		O5	2.61(2)		O9	2.08(3)
	O10	2.70(2)	Te2	O2	1.85(2)	Fe2	O3	1.92(2)
	O6	2.91(3)		O5	1.91(2)		O8	1.97(3)
	O8	2.91(3)		O7	1.93(2)		O5	1.97(2)
Sr2	O12	2.50(2)	Te3	O4	1.81(3)		O7	1.99(3)
	O1	2.61(3)		O3	1.86(2)		O2	2.03(3)
	O9	2.72(3)		O12	1.91(2)		O6	2.12(3)
	O4	2.80(2)		O7	2.64(2)			
	Cl1	2.99(1)	Te4	O9	1.88(3)			
	Cl1	3.03(2)		O1	1.88(3)			
	Cl2	3.04(1)		O10	1.95(2)			
	Cl1	3.11(2)		O9	2.64(2)			

References

1. Alcaraz de la Osa, R.; Iparragirre, I.; Ortiz, D.; Saiz, J. M. The extended Kubelka–Munk theory and its application to spectroscopy. *ChemTexts (Cham)* **2019,** 6 (1), DOI: 10.1007/s40828-019-0097-0.
2. Weil, M. Redetermination of the γ -form of tellurium dioxide. *IUCrData* **2017,** 2 (12), x171757 DOI: 10.1107/S2414314617017576.
